# Exploring functional connectivity at different timescales with multivariate mode decomposition

**DOI:** 10.3389/fnins.2025.1653007

**Published:** 2025-08-28

**Authors:** Manuel Morante, Kristian Frølich, Naveed ur Rehman

**Affiliations:** Department of Electrical and Computer Engineering of Aarhus University, Aarhus, Denmark

**Keywords:** fMRI, Functional Connectivity (FC), multiscale, Multivariate Mode Decomposition (MMD), Multivariate Variational Mode Decomposition (MVMD)

## Abstract

This paper explores an alternative way for analyzing static Functional Connectivity (FC) in functional Magnetic Resonance Imaging (fMRI) data across multiple timescales using a class of adaptive frequency-based methods referred to as Multivariate Mode Decomposition (MMD). The proposed method decomposes fMRI into their intrinsic multivariate oscillatory components through a fully data-driven approach, and enables the isolation of intrinsic neurophysiological activation patterns across multiple frequency bands from other interfering components. Unlike other methods, this approach is inherently equipped to handle the multivariate nature of fMRI data by aligning frequency information across multiple regions of interest. The proposed method was validated using three fMRI experiments: resting-state, motor and gambling experiments. Results demonstrate the capability of the methodology to extract reliable and reproducible FC patterns across individuals while uncovering unique connectivity features at different times scales. In addition, the results evidence the effect of the different task on the spectral organization of FC patterns, highlighting the importance of multiscale analysis for understanding functional interactions.

## 1 Introduction

The brain is a complex system that exhibits highly organized neurophysiological interactions at different timescales (Zalesky et al., 2014; Preti et al., 2017). As a result, brain activity displays highly complex spatio-temporal dynamics (Bolton et al., 2020b; Lurie et al., 2020), and their knowledge is crucial to enhancing our understanding of the brain's function and cognition (Fair et al., 2009; Poldrack et al., 2011; Friston, 2011). Functional Magnetic Resonance Imaging (fMRI) has emerged as a key non-invasive modality for studying brain activity, offering unique insights into neuronal processes through the Blood-Oxygenation-Level-Dependent (BOLD) contrast (Poldrack et al., 2011). This signal reflects variations in oxygenation levels elicited by neuronal metabolic activity, enabling researchers to explore brain functioning indirectly. However, fMRI data is inherently noisy, comprising not only neural signals but also interfering components such as movement, respiratory cycles, and cardiac pulsations (Bianciardi et al., 2009; Bolton et al., 2020a). This complexity requires advanced analysis techniques to reliably interpret the underlying interactions.

Over the past two decades, the study of interactions among neuronal networks has evolved significantly, as discussed by Bolton et al. (2020b). Moving beyond conventional analyses for localizing brain activations, researchers have increasingly focused on understanding the brain's dynamic organization through Functional Connectivity (FC), as envisioned by Friston (2011) more than a decade ago. No doubt, FC analysis has become instrumental in identifying brain network patterns, unraveling cognitive processes, and advancing diagnostics for neurological disorders (Bolton et al., 2020b,a; Calhoun and Adalı, 2012) The recent survey in by Du et al. (2024) offers a good overview of current FC-based approaches for analyzing brain activity. However, they also highlight some of their challenges and limitations. For instance, many conventional approaches rely on assumptions of linearity, stationarity, and independence in the data, which fail to capture the true complexity of brain dynamics, which may compromise the interpretability of results (Guan et al., 2020; Lurie et al., 2020).

Following the current trends discussed by Du et al. (2024), extracting static FC can be broadly categorized into hypothesis-driven and data-driven approaches, as we briefly summarized in Table 1. Hypothesis-driven methods, such as Region of Interest (ROI)-based correlation analysis, have been widely used but require a priori knowledge and may overlook unexpected patterns. In contrast, data-driven methods offer flexibility and adaptability, including matrix decomposition techniques (e.g., Independent Component Analysis, sparse dictionary learning), clustering methods (e.g., hierarchical clustering, k-means), and deep learning models (e.g., CNNs, RBMs, VAEs) (Song et al., 2022; Golestani and Chen, 2022).

**Table 1:** Methods for extracting static functional connectivity from fMRI data.

**Methods for extracting static fuenctional connectivity**			
Hypothesis-driven methods	Region of interest	Static FC extraction	Correlation
			Other metrics
Data-driven methods	Matrix decomposition	Independent component analysis	
		Sparse dictionary learning	
		Nonnegative matrix factorization	
	Clustering	Clustering methods	Hierarchical cluntering
			k-means
			Affinity propagation
		Similarity metrics	High-order features
	Deep learning	Supervised DL	CNN
			RBM
		Unsupervised DL	CNN
			RBM
			DBM
			VAE
	Adaptive ferquency	Static filtering	Hilbert Transform
			Wavelet Coherence
			Iterative filtering
		Mode decomposition	EMD, VMD
			MVMD

**Figure 1:**
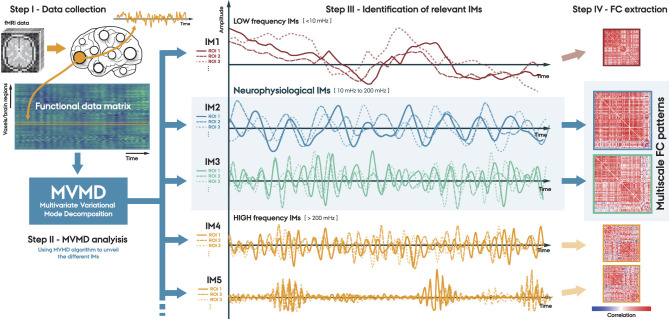
Proposed method. **Step I**. Data collection, where fMRI data is aggregated either at a voxel level or through predefined brain atlases; **Step II**. MVMD analysis, which consists of extracting the Intrinsic Modes (IMs) for each individual; **Step III**. Identify the relevant IMs within the neurophysiological frequency band (10–200 mHz); and **Step IV**. FC extraction associated with the IMs of interest.

This table summarizes current approaches for static FC analysis, extending the categorization reported by (Du et al. 2024) in Figure 1, and highlights our contribution in the context of adaptive frequency-based methods.

While these methods have advanced the field, they are not without drawbacks, as they often struggle with the non-linear and non-stationary nature of fMRI data. For instance, classical frequency-domain transforms, such as Hilbert Transform or Wavelet Coherence (Bolton et al., 2020b), depend on static, predefined bands and implicitly assume stationarity, hindering their ability to deal with individual variability (Yuen et al., 2019). Moreover, recent advances in phase synchronization analysis have revealed that traditional approaches can introduce bias and miss important connectivity patterns occurring at unexpected frequencies as shown by Honari and Lindquist (2022). Similarly, many deep learning methods often rely on static band-pass filtering, uniformly applied across all participants. This preprocessing step inherently assumes that meaningful brain activity occurs within the same frequency ranges for everyone (e.g., 0.01–0.1 Hz), and constitutes a strong oversimplification that fails to account for any task-related or individual variability. On top of that, this limits the view of these approaches to a very specific frequency range, ignoring any other potential information, despite known it carries relevant information (Bolton et al., 2020a; Honari and Lindquist, 2022).

A promising direction—yet often overlook in fMRI studies—lies in adaptive frequency-based methods, which address the limitations of static filtering by decomposing signals into data-driven oscillatory components. For example, mode decomposition techniques, such as the one proposed by Yuen et al. (2019), decompose fMRI signals into intrinsic modes but are limited to univariate analysis. However, (Yuen et al. 2019) primarily focused on voxel-level, and they used only on univariate signal analysis, which ultimately limited its ability to explore the interconnected (multivariate) nature of the fMRI data and its connectivity patterns. In contrast, Multivariate Variational Mode Decomposition (MVMD) Rehman and Aftab, (2019) extends this idea to multivariate data, aligning frequency components across regions and capturing the dynamic interactions between them. This approach overcomes the limitations from Yuen et al. (2019) and those from other conventional FC methods by accommodating the non-linearity, non-stationarity, and multivariate nature of fMRI data, while avoiding rigid assumptions or predefined parameters. These are critical aspects, as the recent study by Honari and Lindquist (2022) has demonstrated over a wide range of simulations, when multiple brain regions are analyzed simultaneously, multivariate approaches significantly outperform univariate methods by ensuring proper mode alignment and avoiding frequency mismatches between decomposed components.

All these observations highlight the need for data-driven signal processing methods for fMRI analysis that can extract relevant frequency information, handle non-linearity and non-stationarity of data, and produce reliable and interpretable results. In response, we propose a novel path for FC analysis in fMRI data that leverages MVMD. MVMD's unique advantage lies in its ability to decompose fMRI signals into their intrinsic multivariate oscillatory components, enabling a comprehensive exploration of brain connectivity across multiple timescales. Additionally, this data-driven approach allows adapting to individual differences without requiring predefined frequency bands or static filters. Unlike conventional approaches that use fixed frequency range, MVMD automatically identifies each individual's intrinsic frequency modes directly from their data, while inherently accommodates the multivariate, non-linear, and non-stationary nature of fMRI data. By aligning frequency information across regions of interest, it provides a robust framework for isolating noise, identifying relevant patterns, and uncovering unique functional interactions (Huang et al., 1998; Rehman and Aftab, 2019). Importantly, MVMD have also been shown to overcome issues such as mode mixing and noise sensitivity compared to other similar Mode Decomposition methods, making it particularly suitable for the analysis of complex, noisy fMRI signals as Honari and Lindquist (2022).

The key contributions of this work are as follows:

1. We introduce an integrated fully adaptive MVMD-based method for fMRI analysis, capable of removing artifacts, filtering noise, and isolating brain activity into fundamental multivariate oscillatory components, while effectively incorporating the non-stationary nature of fMRI data. This framework does not rely on predefined filters or static parameters.
2. We demonstrate the method's ability to uncover reliable and reproducible FC patterns across individuals and experimental conditions, including resting-state, motor, and gambling tasks.
3. We provide new insights into the temporal and spectral organization of brain connectivity, emphasizing the importance of multiscale analysis for understanding functional interactions.

The rest of the paper is organized as follows: Section 2 describes the basis of MVMD, our proposed methodology, the fMRI data, and the experimental design. Section 3 presents the results, highlighting key findings. Section 4 discusses the obtained results and their implications for FC analysis. It also includes a discussion of limitations and potential avenues for future research. Finally, Section 5 concludes with a summary of the study's contributions.

## 2 Materials and methods

Multivariate Variational Mode Decomposition (MVMD^1^) is one of the most popular algorithms used to perform Multivariate Mode Decomposition (MMD) (Rehman and Aftab, 2019). From a general perspective, MMD is a Signal Processing model that assumes a multivariate signal of interest accepts a representation as a linear combination of a small set of amplitude- and frequency-modulated (AM-FM) functions (Huang et al., 1998), each with a well-defined instantaneous frequency shared among all channels (Huang et al., 1998; Rehman and Aftab, 2019).

Formally, given a multivariate signal $x\left(t\right)={\left[x_{1}\left(t\right),x_{2}\left(t\right),\ldots ,x_{C}\left(t\right)\right]}^{T}$, with *C* different channels, the MMD model assumes that the signal arises from a linear combination of *K* intrinsic oscillatory components, {***u***^(1)^(*t*), ***u***^(2)^(*t*), …, ***u***^(*K*)^(*t*)}, referred to as Intrinsic Modes (IMs), as follows:


(1) $$\begin{array}{l} x\left(t\right)=\overset{K}{\underset{k=1}{\sum }}u^{\left(k\right)}\left(t\right)=\overset{K}{\underset{k=1}{\sum }}a^{\left(k\right)}\cos\left(\phi^{\left(k\right)}\left(t\right)\right), \end{array}$$

where $a^{\left(k\right)}\left(t\right)={\left[a_{1}^{\left(k\right)}\left(t\right),a_{2}^{\left(k\right)}\left(t\right),\ldots ,a_{C}^{\left(k\right)}\left(t\right)\right]}^{T}$ and ϕ^(*k*)^(*t*) are the amplitude and the instantaneous frequency of the *k*-th oscillatory component respectively (Rehman and Aftab, 2019). Intuitively, each intrinsic mode behaves similarly to a harmonic function, as they remain relatively close to some common central frequency. Yet, they are still flexible enough to capture non-stationary and non-linear effects by allowing variations in amplitude and frequency as detailed by Dragomiretskiy and Zosso (2014).

Despite its intuitive appeal, performing MMD is not trivial. Consequently, several algorithms have been introduced with different advantages and trade-offs. In this study, we propose to use MVMD, which addresses MMD via a robust optimization-based approach.

### 2.1 Multivariate Variational Mode Decomposition algorithm

Formally, as introduced by Rehman and Aftab (2019), given a multivariate signal ***x***(*t*) containing data from *C* channels, i.e., $x\left(t\right)={\left[x_{1}\left(t\right),x_{2}\left(t\right),\ldots ,x_{C}\left(t\right)\right]}^{T}$, the MVMD algorithm tries to decompose the observed data as a linear combination of *K* principal multivariate oscillations, ***u***^(*k*)^(*t*), as in Equation 1, by solving the following optimization problem:


(2) $$\begin{array}{l} \begin{array}{c} \underset{{\left\{u_{c}^{\left(k\right)}\right\}}_{c,k},{\left\{\omega_{k}\right\}}_{k}}{\text{argmin}}\overset{K}{\underset{k=1}{\sum }}\overset{C}{\underset{c=1}{\sum }}∥\partial_{t}\left[{ŭ}_{c}^{\left(k\right)}\left(t\right)e^{-i\omega_{k}t}\right]{∥}_{2}^{2}\\ \text{s.t.}\;x_{c}\left(t\right)=\overset{K}{\underset{k=1}{\sum }}u_{c}^{\left(k\right)}\left(t\right)\;c=1,2,\ldots ,C, \end{array} \end{array}$$

where ${ŭ}_{c}^{\left(k\right)}$ stands for the Hilbert Transform of $u_{c}^{\left(k\right)}$.

Observe that this particular formulation sets multiple linear constraints, each one corresponding to each particular channel. On the other hand, the main loss function works over the *K* intrinsic modes, where are minimized to remain close to a common central frequency, ω_*k*_.

Solving the optimization task in Equation 2 posses several challenges due to the inherent complexity of the task and the constraints involved. Nonetheless, (Rehman and Aftab 2019) resorted on the classical divide-and-conquer approach, and employed the Alternating Direction Method of Multipliers (ADMM) (Boyd et al., 2011) to effectively integrate multiple constraints. Put succinctly, this approach effectively solves the described optimization task by splitting the problem in multiple easier-to-solve subproblems. The resulting problems are then solved iteratively, producing an estimate of the intrinsic modes. See the original work by Rehman and Aftab (2019) for the particular formulation details and optimization steps.

### 2.2 MVMD and other state-of-the-art MMD algorithms

Although MVMD is one of the most popular algorithms for performing MMD, several alternative methods have been proposed. These include Multivariate Empirical Mode Decomposition (MEMD) (Rehman and Mandic, 2009), multivariate iterative filtering (Cicone and Pellegrino, 2022), and multivariate chirp mode decomposition (Chen Q. et al., 2020). Among these, MEMD stands out as a widely used alternative to MVMD due to some unique features. For instance, MEMD does not require specifying the number of intrinsic modes beforehand (see Supplementary material, Section B for further details), which makes it attractive in cases when it is not possible to find a good estimate for the number of modes.

In essence, MEMD is a data-driven approach that decomposes a multivariate signal into its intrinsic oscillatory components through an iterative greedy process. Similar to MVMD, MEMD aims to extract the same intrinsic modes ***u***^(*k*)^(*t*) as described in Equation 1. However, MEMD employs a distinct iterative sifting process. At each iteration, it estimates a local mean by averaging the maximum and minimum envelopes of the signal. This local mean is then subtracted from the analyzed signal to produce an IM.

Despite its effectiveness in decomposing multivariate signals, MEMD has a significant drawback: it is highly sensitive to noise. This sensitivity can lead to a wide range of problems, including mode mixing, where a single mode contains multiple frequency components (Eriksen and Rehman, 2023). In addition, MEMD is particularly sensitive to noise, as high level of noise may interfere with the detection of the where ${ŭ}_{c}^{\left(k\right)}$ stands for the Hilbert Transform of $u_{c}^{\left(k\right)}$. For all these reasons, MEMD does not appear as a suitable algorithm for fMRI analysis.

### 2.3 Frequency organization of fMRI data

While studies focusing on frequency-related aspects of fMRI are relatively sparse, existing research offers valuable insight into the frequency organization of the fMRI signal and brain dynamics. For instance, Cordes et al. (2001) demonstrates that the frequency contribution to the correlation patterns spans several frequency bands. Similarly, Yuen et al. (2019) investigated the inherent frequency components across different brain locations—in a voxel-wise fashion—yielding similar findings.

Overall, fMRI frequency components comprise a rich spectrum that covers several relevant frequency bands. First, very low-frequency oscillations, much lower than 10 mHz (Power et al., 2011), correspond to trends,or signal drifts. Unlike low-frequency components (Tong et al., 2019), trends and drifts have been consistently been attributed to a combination of physiological fluctuations (Huettel et al., 2009), head motion residuals (Maknojia et al., 2019), and scanner instabilities (Smith et al., 1999). Neurophysiological activation patterns resulting from neuronal activity appear within the range of 10 to 200 mHz (Cordes et al., 2001; Yuen et al., 2019; Lurie et al., 2020) emphasizing the significant contribution of this frequency band to fluctuations related to brain activity, which corresponds with the natural band dominated by the BOLD response.

Additionally, fundamental respiratory oscillations occur around 250 mHz, while the first harmonic of respiration appears around 500 mHz (Frank et al., 2001). Contributions from blood vessels and cerebrospinal fluid pulsations fall within the 400 to 800 mHz band. Similarly, those high-frequency components exhibited significant structured correlations among different brain areas due to the distinct anatomical distribution of the cerebral blood vessels and ventricles (Cordes et al., 2001). Similarly, they also pointed out that cardiac pulsations can spread to lower frequencies due to aliasing, appearing as additional interfering structured components, which complies with observations by Soon et al. (2021).

### 2.4 Proposed approach: multiscale functional connectivity using MVMD

In this study, we propose a new method for analyzing FC using MVMD. The Figure 1 illustrates the main steps of our proposed approach:

- **Step I. Data collection**. The first step involves collecting data from each individual. We can perform this step at the voxel level or over some set of ROIs, using an appropriate brain atlas.
- **Step II. MVMD analysis**. We perform MVMD analysis on the data collected using a well established algorithm (see text footnote ^1^). This analysis obtains the intrinsic oscillatory components (IMs) associated with each individual.
- **Step III. Identification of the relevant IMs**. We identify the relevant IMs by examining their corresponding central frequencies. Specifically, we focus on the components within the neurophysiological frequency band 10–200 mHz, as discussed in Section 2.3.
- **Step IV. Functional Connectivity (FC) extraction**. We can use the obtained multivariate IMs associated with each particular frequency band to uncover the FC at various timescales, providing a complete multiscale FC representation of the fMRI data.

### 2.5 fMRI data, experimental description, and preprocessing

In this study, we investigated three experiments from the WU-Minn Human Connectome Project (HCP) (Van Essen et al., 2013). Specifically, we selected the resting state, motor, and gambling experiments from the HCP repository^2^. In each experiment, we randomly selected 100 healthy participants aged 22 to 35 years.

The first experiment was resting-state, where participants were instructed to remain as still as possible during the scan, with eyes open with relaxed fixation on a projected bright cross-hair on a dark background and presented in a darkened room^3^. We chose this experiment because resting-state data is widely used for FC analysis, often providing reliable results.

The motor experiment followed a standard block paradigm, where a visual cue asked the participants to tap their left or right fingers, squeeze their left or right toes, or move their tongue. Each movement block lasted 12 s and was preceded by a 3-s visual cue. Additionally, there were two 15-s fixation blocks. We chose this simple experiment because the activation patterns and neuronal networks involved are well documented (Morante et al., 2020), facilitating the evaluation of the results.

Last but not least, the gambling experiment followed a random block paradigm, where participants tried to guess if a randomly generated number between 1 and 9 was either higher or lower than 5. There are two main reasons why we investigated this additional task-related experiment. Firstly, this experiment has been well studied, making the evaluation process easier. The second reason is that, unlike the motor experiment, the gambling experiment's paradigm is unpredictable, i.e., the guesses of the participants cannot be determined a priori. This randomness adds an unpredictable effect to the responses, increasing the variability in the data and allowing for a more robust and consistent analysis. Additionally, we expect the level of arousal and effort for this experiment to be higher than for the other two experiments.

#### 2.5.1 Preprocessing

We obtained the fMRI data directly from the HCP repository (see text footnote ^2^). The datasets used were collected using a 3T scanner with a repetition time (TR) of 720 ms. The specific descriptions of the experimental procedures and acquisition parameters are detailed in the HCP imaging protocols (see text footnote ^3^). In particular, we analyzed the data with minimal preprocessing steps, including motion correction and spatial normalization. Finally, on top of the standard preprocessing pipeline already applied by the HCP (Barch, 2013; Van Essen et al., 2013), we further smoothed each brain volume with a 4-mm FWHM Gaussian kernel.

As discussed in the introduction, our proposed methodology does not require any further preprocessing steps, such as static temporal filtering or source separation, to uncover the natural oscillatory components of the fMRI data. Unlike, for example, Yuen et al. (2019), who explicitly required static temporal filtering.

#### 2.5.2 Selected regions of interest

We divided the brain into several ROIs using the Automated Anatomical Labeling (AAL) atlas (Tzourio-Mazoyer et al., 2002). Although the AAL atlas maps the entire brain, we only analyzed cerebral regions, which resulted in 90 ROIs. Following the recent module-based network organization proposed by Parente and Colosimo (2020), we grouped these 90 ROIs into seven functional modules. For the selected 90 ROIs, we extracted the related time series using Nilearn toolbox^4^. Finally, we removed the mean value from each ROI.

For completeness, Supplementary Table 1 in the Supplementary material contains detailed information regarding the ROIs selected from the AAL and its modules, including labels and network organization.

Additionally, only for the motor experiment, we considered an extra 5 ROIs for the analysis of the time courses associated with the different parts of the motor cortex. For extracting these areas, we used the same motor templates for separating these motor ROIs as, for example, the one implemented by Morante et al. (2021).

#### 2.5.3 Parameter selection for MVMD

MVMD is a parametric algorithm that requires setting two parameters: the number of intrinsic modes, *K*, and the regularization parameter α, which essentially controls the bandwidth of each IM. In this study, we conducted a small exploratory study to determine the optimal value for these parameters in terms of energy and signal reconstruction, and we found that *K* = 10, and α = 1, 000 produced good signal reconstruction. All the details regarding this evaluation are discussed in the Supplementary material.

## 3 Results

In this study, we extracted the intrinsic oscillatory components from all three fMRI experiments evaluated, following the procedure described in Figure 1. Then, we estimated the FC patterns associated with each IM among all the different participants. Finally, we conducted a quantitative analysis of the obtained FC patterns to evaluate their reproducibility among participants.

### 3.1 Intrinsic mode extraction using MVMD

**Figure 2:**
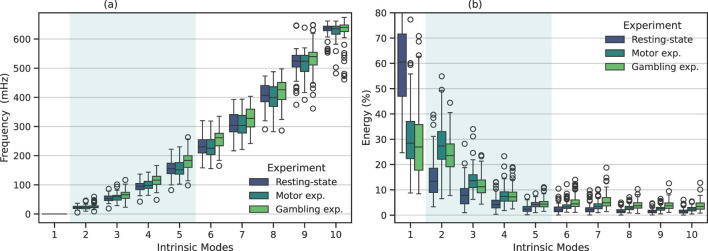
Frequency and energy distribution associated with each IM using MVMD. The boxplot depicts the corresponding results among all the studied participants for the resting-state, motor, and gambling fMRI experiments. The shadowed area highlights the intrinsic modes whose frequency appears within the neurophysiological band. **(a)** Frequency distribution for MVMD. **(b)** Energy distribution for MVMD.

We examined the intrinsic modes obtained using MVMD among all the different experiments. Figure 2 illustrates the central frequencies, ω_*k*_, (a), and relative energy contribution (b) of the IMs for all the studied participants. We calculated the relative energy contribution associated to each mode as the ration between the energy of the *k*-th mode, say *E*_*k*_, and the total energy of the signal, *E*_*tot*_, i.e., *E*_*k*_/*E*_*tot*_. Each colored box depicts the results associated with each studied experiment. For convenience, the shadowed area highlights the IMs within the neurophysiological bandwidth.

Regarding the intrinsic modes, we observed that the first IM exhibited a dominant frequency, centered around zero. Similarly, this mode showed a higher relative energy contribution among all participants. IMs 2 to 5 appeared within the neurophysiological frequency range. This range includes typical brain activity frequencies related to several cognitive and neurophysiological processes (Cordes et al., 2001). Table 2 shows the average central frequencies for each IM among participants and their corresponding bandwidths. We found that those results were relatively consistent among participants and experiments.

**Table 2:** Average frequency, $\bar{f}$, and their corresponding average bandwidth (BW) for the different studied experiments for MVMD.

	**Resting-state**		**Motor**		**Gambling**	
	$\bar{f}$	**BW (mHz)**	$\bar{f}$	**BW (mHz)**	$\bar{f}$	**BW (mHz)**
Mode 1	0	6.8 ± 1.0	0	21.6 ± 1.5	0	26.3 ± 1.5
Mode 2	23	14.3 ± 1.9	23	36.5 ± 4.1	26	44.9 ± 4.3
Mode 3	54	15.0 ± 2.2	56	40.5 ± 1.9	66	50.8 ± 2.3
Mode 4	96	15.9 ± 2.7	99	43.0 ± 3.1	115	53.0 ± 2.9
Mode 5	156	16.9 ± 2.7	157	47.6 ± 3.9	182	57.4 ± 3.6
Mode 6	232	16.5 ± 2.8	231	50.6 ± 3.6	257	57.0 ± 3.8
Mode 7	310	17.2 ± 3.1	308	50.8 ± 3.9	330	57.6 ± 4.1
Mode 8	407	18.1 ± 2.5	400	54.1 ± 4.5	419	61.6 ± 4.4
Mode 9	521	18.3 ± 2.4	514	56.6 ± 3.9	533	63.8 ± 4.0
Mode 10	632	17.4 ± 2.3	627	53.8 ± 5.2	627	56.8 ± 7.4

The notation ± indicates the standard deviation among all studied participants. Formally, for the *k*-th intrinsic mode, we calculated the average central frequency, ${\bar{f}}_{k}$ as the arithmetic mean of the central frequencies of all participants, i.e., ${\bar{f}}_{k}=\frac{1}{N}\overset{N}{\underset{n=1}{\sum }}\omega_{k,n}$, where *N* is the number of participants and ω_*k, n*_ is the central frequency of the *n*-th participant for the *k*-th mode.

The remaining modes spanned across high frequencies. As discussed in Section 2.3, signals within this frequency range originate from a mixture of different interfering components. These components include those induced by respiration movements, heartbeat, and cerebrospinal fluid pulsations. Notably, mode 6—approximately centered at 250 mHz—appears close to the primary respiratory-related harmonic, while mode 9—with a central frequency of ~520 mHz– closely coincided with the first harmonic of the cardiac pulsations (Cordes et al., 2001; Yuen et al., 2019).

Regarding the relative energy contribution among IMs, illustrated in Figure 2b, we observed that, in general, modes with lower frequencies exhibited higher energy, while those with higher frequencies had decreasing energy contributions. Specifically, we observed that the first IM captured residual trends, characterized for having a central frequency around zero (see Table 2). This indicates that this IM is primarily capturing slow signal drifts (Huettel et al., 2009; Power et al., 2011), rather than former oscillatory components. In this way, unlike other general low-frequency components (Tong et al., 2019), the first IM primarily appeared to capture trends and slow signal drifts (Huettel et al., 2009; Power et al., 2011; Maknojia et al., 2019), which have consistently been attributed to a combination of physiological fluctuations (Huettel et al., 2009; Power et al., 2011), head motion residuals (Maknojia et al., 2019), and scanner instabilities (Smith et al., 1999). In contrast, the high-frequency components, over the neurophyisiological band, which are associated to respiratory and cardiac harmonics, showed comparatively lower energy contributions. The reason for this is that these high-frequency components are more localized (e.g., in the cerebrospinal fluid or blood vessels), resulting in a relatively smaller contribution to the overall fMRI signal, compared to other physiological components, such for example head movements, that affect the full brain.

#### 3.1.1 Comparison between fMRI experiments

Overall, Figure 2 shows that the intrinsic modes obtained from the different fMRI experiments display similar central frequencies and energy contributions, as they follow a similar trend. However, a closer examination of the energy and frequency distributions revealed some interesting differences.

For instance, in the resting-state experiment, the majority of the energy is concentrated at low frequencies, where the first mode appeared as the most energetic component. In contrast, task-related experiments—although their trend is similar—showed slightly different behavior: the first component was relatively less dominant, whereas the neurophysiologically relevant modes (IMs 2–5) consistently exhibited higher energy levels compared to the resting-state condition. Particularly, IMs 2 and 3 showed significantly higher energy levels, followed by IMs 4 and 5, which were also elevated compared to the resting-state results. In other words, neurophysiological components associated with task-related activity displayed higher energy contributions than those at rest. This observation is intuitively consistent with the nature of these experiments, which involve cognitive tasks such as motor and gambling tasks that require increased neuronal activity.

On the other hand, when focusing on the components outside the neurophysiological band, we also observed some relevant differences. For example, modes 6 and 7 in the gambling experiment exhibited higher central frequencies than the other experiments. Similarly, when examining the energy distribution, we observed a significant increase in the energy associated with these modes compared to resting-state. From Section 2.3, we understand that the mode 6 falls within the physiological range associated with the first respiratory harmonic. Therefore, both higher frequency and energy indicate a faster respiratory rhythm. In this sense, these results evidenced that our proposed approach is able to capture and separate both, brain and physiological changes.

### 3.2 Overview of intrinsic modes from MVMD

#### 3.2.1 IMs from the resting-state experiment

**Figure 3:**
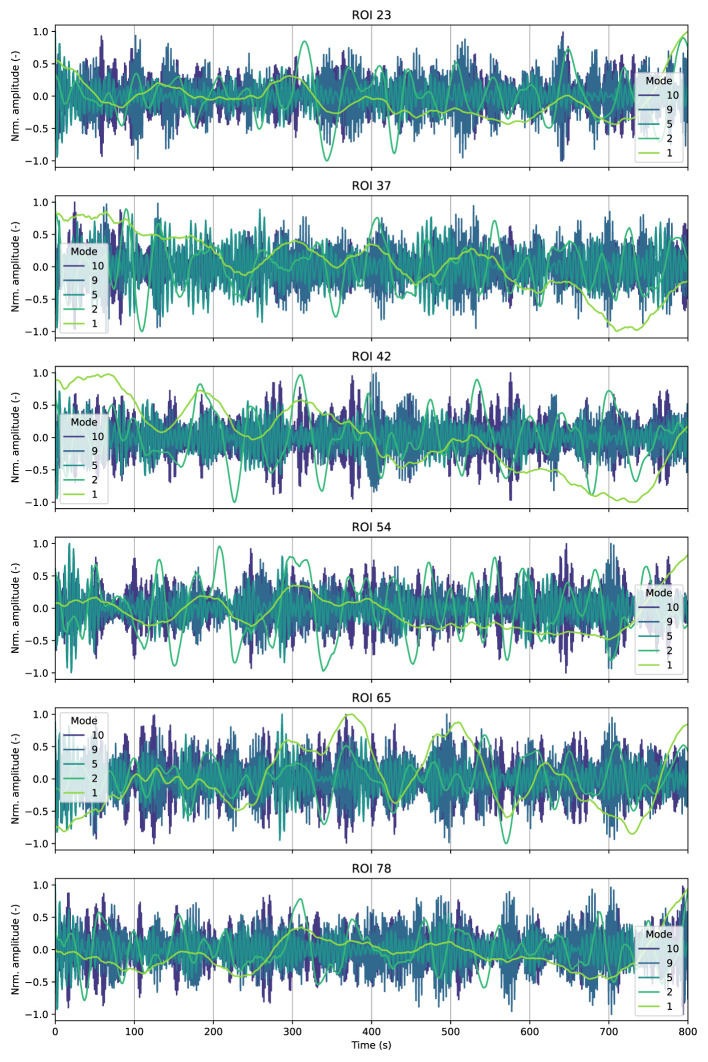
Time activation patterns associated with some IMs among several randomly selected ROIs from a randomly selected individual. The results correspond to the analysis of the resting-state experiment.

We visualize the activation patterns associated with some IMs for different ROIs. The idea is to better illustrate the behavior of these IMs as well as gain a deeper understanding of their physiological meaning. Figure 3 displays the IMs within some randomly selected ROIs for a randomly selected participant. We also observed that the first mode captured low-frequencies trends and signal drifts, likely due to motion residuals or scanner inestabilities (Power et al., 2011; Maknojia et al., 2019; Smith et al., 1999), which is also reflected in the results in Figure 3.

Even though we observed high levels of consensus among all participants on frequency and energy distributions, a closer examination of the specific time activation patterns associated with this mode in Figure 3, revealed that these patterns varied among participants, which indicates that these low-frequency components are highly individual-dependent. Furthermore, intrinsic modes with high frequencies, including those in respiratory and cardiac bandwidth, also showed considerable individual variability.

#### 3.2.2 IMs from the motor experiment

In contrast to the resting state, the motor experiment allowed us to perform a more comprehensive analysis of the IMs and their neurophysiological meaning. First, we selected the primary visual cortex (ROI 43 and 44) for visual responses. In addition, we further divided the motor cortex ROI into five additional motor-related ROIs for the different motor areas, corresponding to the right/left hands, right/left feet, and the tongue (Morante et al., 2020).

The motor experiment follows a conventional block paradigm, therefore, we have access to the canonical task-related components, i.e., the time activation patterns that are obtained using the classical convolutional model with the canonical Hemodynamic Response Function (HRF) (Power et al., 2011). These components serve no particular purpose for our proposed approach, but we will use it as a reference to better illustrate IMs' behavior.

**Figure 4:**
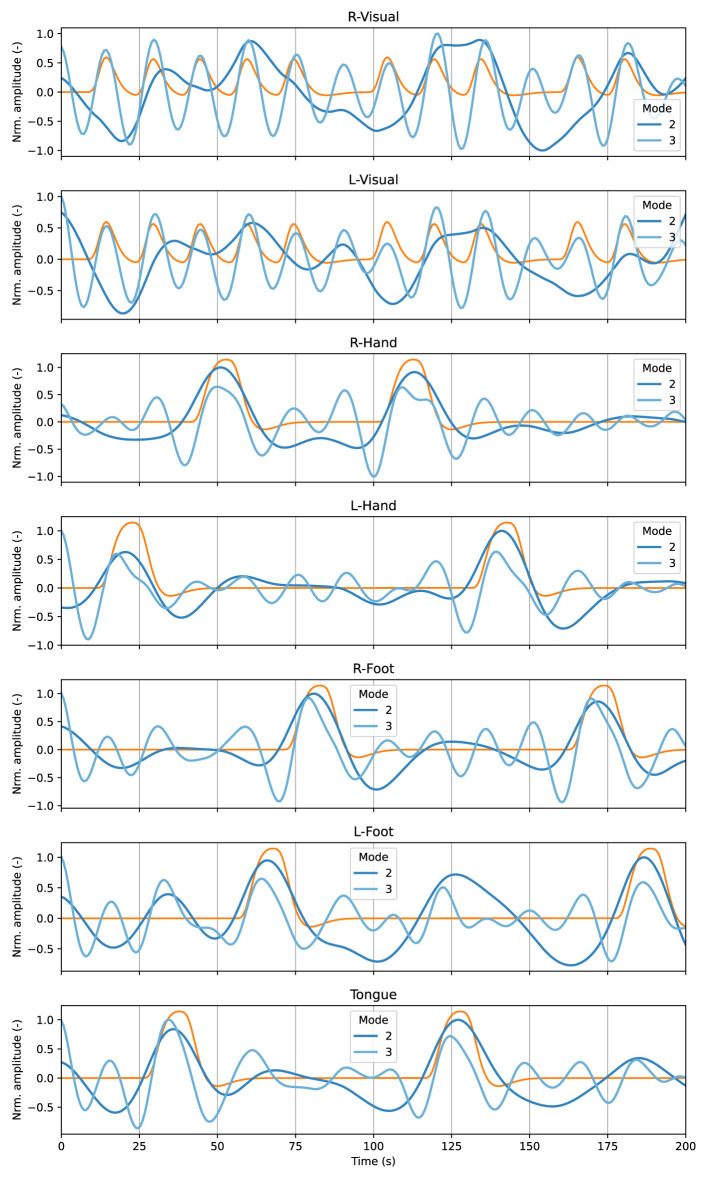
Average time courses among all the studied participants associated with the first two neurophysiological IMs (2 and 3) from the results of MVMD (blue lines), and the canonical task-related components (orange lines) for the most relevant ROIs associated with the motor experiment.

Figure 4 illustrates the average time courses for the relevant ROIs associated with the motor experiment among all the participants from the MVMD algorithm. The blue-colored lines correspond to the average of the specific time activation patterns for IMs 2, 3 and 4 among all the studied participants. Finally, the orange lines in Figure 4 represent the canonical task-related component expected within each main ROI. In this case, we focused only on the task related components, as we aim to compare them with the canonical task-related component. Higher frequency modes are not included as they exhibit similar behavior as the ones from Figure 3, as well as strong individual variability, and renders their average meaningless.

Those results showed that IM2 and IM3 effectively capture information related to the expected brain activation patterns within their corresponding ROIs of interest. For instance, IM 2 closely aligns with the block-related activity from the motor cortex ROIs. On the other hand, IM 3, which exhibits a higher central frequency (see Table 2), encodes the visual cue associated with the motor task and fixation.

### 3.3 Multiscale static functional connectivity using MVMD

**Figure 5:**
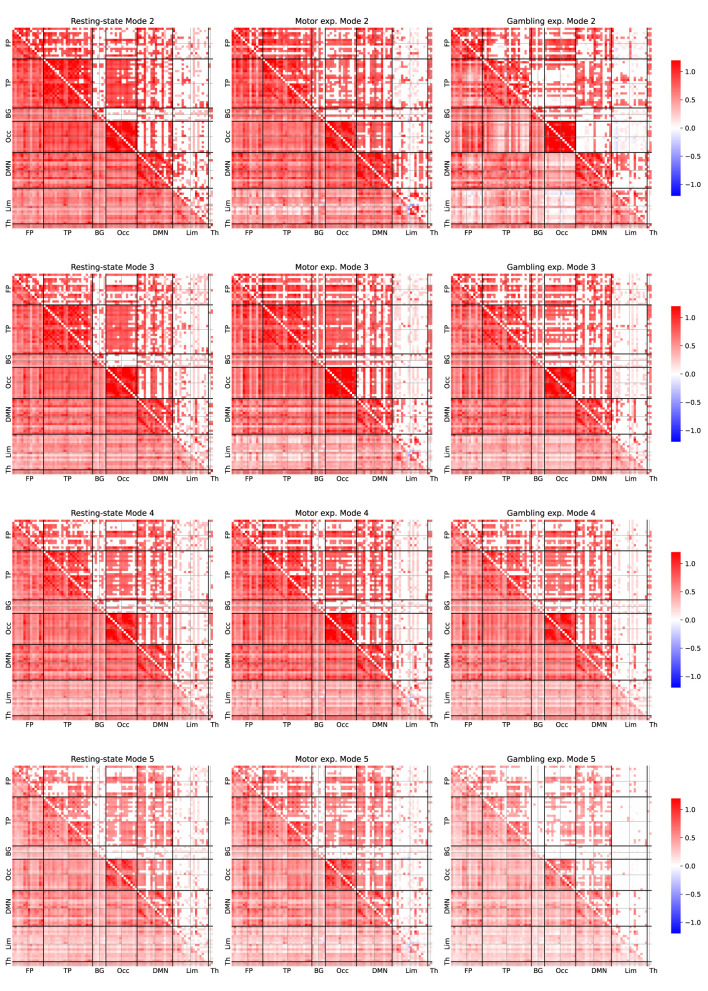
Average FC patterns for the IMs 2, 3, 4, and 5 for the three studied fMRI experiments. The FC patterns were estimated by averaging across 100 participants. Person's correlation coefficients were Fisher-Z transformed. The lower diagonal part shows all the averaged correlation coefficients. The upper diagonal only displays significant correlation coefficients compared to the null dataset from a permutation-based *t*-test corrected with a false positive rate adjusted to *p* ≤ 0.001.

Figure 5 depicts the average FC patterns associated only with neurophysiological modes. We obtained those FC patterns by averaging them across all participants, as they exhibited high similarities. Each row corresponds to a particular mode, and each column contains different experiments. For all comparisons, we performed a statistical test with respect to a null dataset generated from each particular decomposition by randomly mixing the temporal samples of the IMs. Pearson's correlation coefficients were Fisher-Z transformed. The lower diagonal of each connectivity matrix displays the average correlation coefficients, while the upper diagonal shows only the correlation values that also exhibited significant activation compared to the null data derived from a permutation-based *t*-test corrected for false discovery rate adjusted to *p* < 0.001, as used by Romanello et al. (2022), as provide robust statistical analysis without assumptions about the underlying data distributions. For convenience, we arranged the ROIs according to the leading module (left and right), following the order reported in Supplementary Table 1.

Overall, by examining Figure 5 we observed some overlap in connectivity patterns across IM 2–5 within each experiment. To further investigate this observation, we examined the FC patterns associated with IM 1 and IM 6–10 (see Supplementary Figures 3, 4). Notably, the strong consistent connectivity patterns observed across IM 2–5 were largely absent in the higher-frequency components (IM 6–10).

#### 3.3.1 Reproducibility of the FC maps among participants

Reproducibility refers to the ability of methods to consistently detect consistent activity within the expected ROIs among different experimental realizations (Varoquaux et al., 2010; Morante et al., 2020). Figure 5 contains the average FC patterns for each mode. However, this figure does not provide any additional information regarding the reproducibility of these results among participants. Consequently, we examined the reproducibility of the reported results. In this regard, this step differs from other approaches (Amico and Goñi, 2018), in that it constitutes a necessary step to ensure results consistency.

Therefore, we conducted a study to assess FC patterns reproducibility among participants. This study aimed to demonstrate how different FC patterns behaved across participants. In particular, we studied the individual reproducibility of the static FC patterns associated with each IM for the three considered fMRI experiments.

For completeness, we evaluated the reproducibility of FC patterns across all IMs, including those from outside the neurophysiological band. To this end, we calculated the Pearson's correlation obtained from all the possible pairs of comparisons across all participants. We want to emphasize that this step was critical in ensuring the validity of our findings, as it allowed us to confirm that the FC patterns associated with each IM were consistent across all participants, providing a solid foundation for our findings.

**Figure 6:**
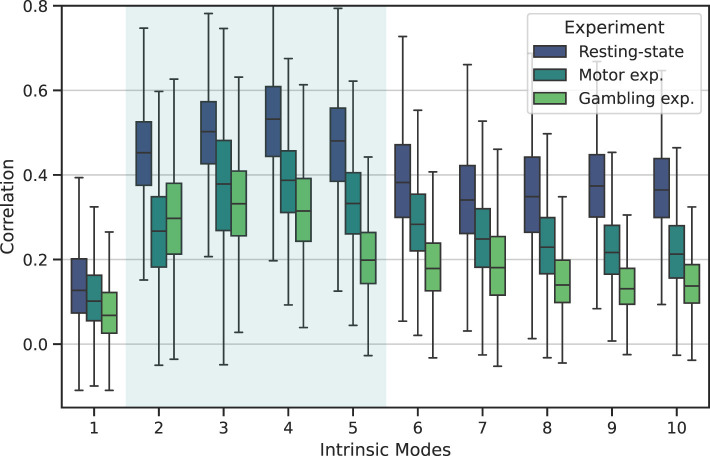
Reproducibility of the static FC patterns associated with each intrinsic mode for the three considered fMRI experiments using MVMD. The boxplots depict the Pearson's correlation values among all the possible individual pair comparisons across all the participants.

Figure 6 illustrates the similarity of the FC associated with each mode using MVMD for the three studied experiments. The boxplots depict the results obtained across all pair comparisons for all participants, for each experiment separately.

Upon examination, we observed that all experiments followed a similar trend. Specifically, we observed that, although each experiment exhibit slightly difference reliability values, within each experiment, the neurophysiological IMs (IM 2–5) consistently exhibited higher reproducibility than the rest of the components (IM 1, IM 6–10).

## 4 Discussion

In this study, we explored the FC patterns associated with the intrinsic modes (IMs) extracted using MVMD across different fMRI experiments. Our results demonstrated that MVMD effectively unveiled the inherent oscillatory components that closely matched the natural characteristics of the fMRI data.

The study of the FC patterns associated with those IMs also provided insightful information regarding the brain's behavior at different timescales. We also observed differences among the studied experiments, which underscores the relevance of examining various temporal scales to gain a comprehensive view of brain function (Chen J. E. et al., 2020).

Our results provide empirical evidence that MVMD effectively extracts the intrinsic oscillatory components of fMRI data. Specifically, we observed that the IMs obtained from MVMD aligned closely with the expected behavior of fMRI signals, as their frequency bands and energy distribution adhered to established characteristics (see Section 2.3).

Furthermore, when we examined the reproducibility of the FC patterns associated with these IMs in Figure 6, we noted a similar trend across all experiments: modes within the neurophysiological band exhibited the highest reproducibility. In particular, IM 3 and IM 4 demonstrated remarkable consistency, compared to those outside the neurophysiological band.

The behavior of the results reported in Figure 6 were expected. We aim to uncover common neurophysiological interactions, so we anticipated components within the neurophysiological band producing similar FC patterns to those outside this frequency band. For instance, we observed that the first IM, despite having the highest energy, exhibited the lowest reproducibility among participants and experiments. Because the first intrinsic mode captures individual trends and motion residuals, which are highly individual-specific (Power et al., 2011).

Additionally, although the discussed behavior appears consistently across experiments, Figure 6 also revealed some differences between experiments. In this study, we estimated reproducibility separately for each experiment. Therefore, we hypothesize that the observed differences may reflect inherent variations in FC characteristics between resting-state and task-related experiments. However, we acknowledge that the nature of these differences remains unclear: one possible explanation is that neurophysiological IMs tend to exhibit higher consistency during resting-state, while task-related experiments may contain greater variability, likely due to individual differences in task performance. Alternatively, those differences could stem from limitations of MVMD itself, as task-related experiments may contain higher level of noise and interfering motion residuals, leading to systematic differences between task-related and resting-state conditions.

On the other hand, we observed that the average activation patterns for neurophysiological IMs for some ROIs comply with the expected canonical task-related activation patterns from the motor experiment as illustrated in Figure 4. In contrast, we observed a large difference between ROIs for IMs outside the neurophysiological band, indicating that those components are more individual-specific, which also appeared within the resting-state experiment (see Figure 3).

Regarding the high-frequency intrinsic modes, above the neurophysiological band, although those showed lower reproducibility, some of them still demonstrated a relatively large similarity. These findings indicate that certain interfering physiological components also produce structured and consistent FC patterns among individuals. These results suggest that high-frequency modes associated to cardiac or respiratory harmonics exhibits their own particular characteristics, which is consistent with the findings by Chen J. E. et al. (2020).

### 4.1 Analysis of IMs between fMRI experiments

The central frequency and energy distributions for MVMD in Figure 2 displayed similar trends across all experiments. However, closer examination revealed some differences between the experiments. In the gambling experiment (Figure 2), for instance, IMs 5 and 6 exhibited significantly higher central frequencies than those in other experiments.

Similary, IMs 6 and 7 also contributed significantly to the fMRI signals in the gambling experiment. These findings indicate faster physiological rhythms during the gambling experiments, which we attribute to higher arousal levels. The higher central frequency, especially in IM 6, points to faster respiratory cycles. IM 9 also showed increased energy, consistent with the first respiratory harmonic. However, when compared to the other experiments, this difference was not statistically significant.

Low-frequency IMs revealed additional differences in energy distributions across fMRI experiments. In the resting-state experiment, energy appeared concentrated primarily in the first IM, then rapidly shifted to IMs 2 and 3 before vanishing at higher frequencies. In contrast, in task-related experiments, the contribution of the first IM was considerably lower, while IMs 2-4 exhibited consistently higher energy. This pattern suggests that task-related experiments involve increased energy demands within the neurophysiological band and reflect the heightened neuronal activity required to perform cognitive tasks.

These results also align with previous research indicating that cognitive engagement leads to stronger BOLD signal changes due to localized oxygen consumption and vascular responses (Power et al., 2011). Consequently, our findings underscore the fundamental contrast between minimal cognitive engagement during resting-state scans and more complex neuronal activation patterns in task-based experiments, which ultimately leads to higher overall energy contributions within these intrinsic modes.

### 4.2 Neurophysiological multiscale FC patterns

Regarding the FC study, Figure 5 shows the average FC patterns associated with neurophysiological IMs from MVMD. Overall, we identified some similarities between the experiments. The presence of similar connectivity patterns across multiple frequency bands likely reflects common underlying anatomical connectivity structures that support brain activity across the neurophysiological spectrum. These findings align with previous static FC studies (Bolton et al., 2020b) that the presence of similar connectivity patterns across multiple frequency bands likely reflect a common underlying connectivity structure, large-scale nets of interconnected neurons that mediates and support the signaling between brain regions (Hermundstad et al., 2013). Furthermore, the similarity of FC patterns among participants declined with increasing frequency (Supplementary Figure 4), supporting our hypothesis that higher-frequency components are more likely driven by physiological noise rather than neurophysiological processes.

On the other hand, the FC patterns associated with IM 1 presented a more complex behavior (Supplementary Figure 3), exhibiting some consistency across participants with partial overlap to IM 2–5 patterns. However, it also exhibits large individual variability as shown in Figure 6, this indicates that despite having some common patterns, it also contains large individual differences, which reflect the interplay and mixture of several components, such as scanner instabilities and motion artifacts (Maknojia et al., 2019).

Upon closer inspection, we also uncovered some differences, which provide further opportunities to explore the underlying brain mechanisms. For instance, IM 2 exhibited extensive activity across several brain networks, including the default mode network (DMN), the temporal and occipital modules. When we analyzed the resting-state patterns, we observed more significant correlations between the occipital and temporal modules than in task-related experiments, suggesting broader engagement of neural networks such as the attention network.

The motor task, on the other hand, showed a higher number of significant correlations between the occipital region and the DMN. In contrast, the gambling experiments showed fewer significant correlations. This finding suggests a specific involvement of visual control processes, aligning with FC dynamics reported in other task-related experiments (Branzi et al., 2022).

Furthermore, during the gambling experiment, we noted strong left-hemisphere correlations within the limbic module. This lateralization may reflect emotional responses to gambling tasks (Wu et al., 2022). The DMN and the occipital module also exhibited significant correlations, highlighting the interplay between intrinsic processing and visual input during this condition.

Regarding IM 3, we observed more consistent patterns across experiments. Task-related paradigms consistently showed a significant connection between the frontoparietal and temporal modules, marked by left-lateralization that weakened in the resting-state condition.

Finally, IM 4 revealed minimal differences among experiments, implying that higher-frequency neurophysiological IMs exert relatively low influence on task-related activity. We hypothesize that other paradigms designed to challenge higher cognitive functions might uncover distinct connectivity patterns at these frequencies. Future research should explore whether more demanding tasks or additional cognitive loads could alter high-frequency FC contributions.

### 4.3 Limitations and future work

As with any other study, there are some limitations: (1) We analyzed three fMRI datasets with similar imaging protocols. Future experiments with a broader range of datasets and conditions would help us to fully understand the applicability of the proposed approach. (2) Exploring dynamic FC aspects using MMD was beyond the scope of this study. A thorough examination of connectivity dynamics following the proposed approach and how they relate to other dynamic FC approaches could be explored in future work. (3) We focused on a single brain atlas. A comparison with other alternative brain atlases could also provide additional insights. (4) Our study focused on the ROI level. Exploring the voxel-level application of MFC could yield fine-grained insights into brain FC. (5) The current MVMD algorithms are applied individually. More advanced algorithms tailored to fMRI data and group-level strategies that integrate information from multiple participants at the same time could be investigated in future research.(6) In this study, we analyzed the three different fMRI experiments separately. As a direction of future work, we suggest performing a more comprehensive joint analysis, as this would enable more robust cross-experimental comparisons.

## 5 Conclusions

In this study, we introduce a novel method for extracting neurophysiological and functional information from fMRI data across multiple timescales using Multivariate Variational Mode Decomposition (MVMD). To the best of our knowledge, this is the first time applying such an analysis to fMRI data. Our method differs from prior studies, such as Yuen et al. (2019), by addressing the non-linear, non-stationary, and multivariate nature of fMRI data while incorporating individual-specific characteristics in a data-driven manner, without the need for predefined static filters or source separation.

Through the analysis of three distinct fMRI experiments, we demonstrated that MVMD effectively extracts intrinsic modes (IMs) from fMRI data. These IMs complied with fMRI frequency organization and provided meaningful and reproducible FC patterns across multiple timescales. Furthermore, the study of these connectivity patterns highlighted the interconnected roles of various brain networks at different timescales, and showed interesting differences between the fMRI experiments. Thus, our method offers a more comprehensive understanding of fMRI dynamics and network interactions (Preti et al., 2017).

## Data Availability

The original contributions presented in the study are included in the article/Supplementary material, further inquiries can be directed to the corresponding author.

## Footnotes

^1^Open-source code for MVMD available for Python (https://github.com/Dmocrito/mvmd) and MATLAB (https://se.mathworks.com/matlabcentral/fileexchange/72814-multivariate-variational-mode-decomposition-mvmd).

^2^Human Connectome Project: https://www.humanconnectome.org/.

^3^HCP 3T Imaging Protocol Overview: http://protocols. humanconnectome.org/HCP/3T/imaging-protocols.html.

^4^Nilearn: https://nilearn.github.io/stable/index.html.
