# Supplementary material for: Exploring functional connectivity at different timescales with multivariate mode decomposition
Source: Front Neurosci. 2025 Aug 28;19:1653007. doi: 10.3389/fnins.2025.1653007 (PMC12423033; doi:10.3389/fnins.2025.1653007)
Supplement: Supplementary file 1 [file Data_Sheet_1.pdf]

Supplementary Material

Multiscale Functional Connectivity: Exploring the brain functional connectivity at different timescales.

Manuel Morante, Kristian Frølich, and Naveed ur Rehman

Contents

1 Selected regions of interest **1**

2 Parameter selection of MVMD **1**

3 FC patterns associated with nonneurophyisiological IMs **3**

1 Selected regions of interest

Table 1 contains information regarding the ROIs selected from the AAL (Tzourio-Mazoyer et al., 2002) and its modules as described by Parente and Colosimo (2020).

2 Parameter selection of MVMD

Multivariate Variational Mode Decomposition (MVMD) requires two parameters to be manually tuned: the number of modes K and the regularization parameter α. The parameter K naturally represents the number of components we expect to observe in the data. On the other hand, the parameter α is a regularization parameter introduced during the optimization that imposes a constraint over the bandwidth of each IM. In this way, a higher value results in intrinsic modes with narrower frequency bands. Since the values of these parameters are unknown a priori, we performed a cross-validation over the 100 participants to identify the optimal values for these parameters. We did this by varying the number of intrinsic modes, K, in the interval of

ROI	Region	Module	
7/8	Frontal middle		
9/10	Frontal middle orbital		
11/12	Frontal inferior opercular		
13/14	Frontal inferior triangular	FP	Fronto-Parietal
15/16	Frontal inferior orbital		
59/60	Parietal superior		
61/62	Parietal inferior		
1/2	Precentral		
17/18	Rolandic operculum		
19/20	Supplementary motor area		
29/30	Insula		
57/58	Post-central		
63/64	Supramarginal	TP	Temporo-Parietal
69/70	Paracentral lobule		
79/80	Heschl		
81/82	Temporal superior		
83/84	Temporal pole superior		
89/90	Temporal inferior		
71/72	Caudate		
73/74	Putamen	BG	Basal Ganglia
75/76	Pallidum		
43/44	Calcarine		
45/46	Cuneus		
47/48	Lingual		
49/50	Occipital superior	Occ	Occipital
51/52	Occipital middle		
53/54	Occipital inferior		
55/56	Fusiform		
3/4	Frontal superior		
23/24	Frontal superior medial		
31/32	Cingulum anterior		
33/34	Cingulum middle	DMN	Default Mode Network
35/36	Cingulum posterior		
65/66	Angular		
67/68	Precuneus		
85/86	Temporal middle		
5/6	Frontal superior orbital		
21/22	Olfactory		
25/26	Frontal medial orbital		
27/28	Rectus	Lim	Limbic
37/38	Hippocampus		
39/40	Para-hippocampus		
41/42	Amygdala		
87/88	Temporal pole middle		
77/78	Thalamus	Th	Thalamus

Table 1: Summary of the selected ROIs organized according to the functional modules described by Parente and Colosimo (2020). The numeric label refers to the 90 ROIs associated with the AAL from Tzourio-Mazoyer et al. (2002), where odd and even numbers correspond to the right and left hemispheres, respectively.

$6-12$ and analyzing the resulting center frequencies and energy distribution. Figure 1 displays the results from this study.

The first column in Figure 1 shows that at least 8 IMs were needed to separate the intrinsic modes adequately. For instance, when looking at the case $K = 6$, we observed that modes 5 and 6 exhibited a larger variance, indicating that the high-frequency components were not separated correctly. Therefore, $K = 8$ constitutes a suitable lower bound for K. The second column in Figure 1 shows the relative energy of the modes, where we observed that the energy present in the modes is decreasing for the higher modes. This means using more than 10 modes will contain mostly noise or residual information, whereas high-frequency modes split into more and more components that barely contribute to the signal. For instance, the results for $K = 12$, where the modes with the highest frequencies exhibited minimal contributions to the relative energy of the signal. Consequently, after evaluating all these arguments, we decided to set the value to $K = 10$, which is big enough to provide enough components and, at the same time, preserves the most information.

Regarding the second parameter, α, associated with MVMD, we selected this parameter similarly to K. For this case, we explored the values of this parameter within the interval $500 - 1500$. The resulting plots from this study can be seen in Figure 4. The first column shows that a higher value of α results in more narrow bands for the corresponding IMs within the neurophysiological band of $10 - 200$ mHz, which is preferable. Looking at the second column in Figure 4, we observed minimal changes in the relative energy distribution. This result was expected Rehman and Aftab (2019), as α is a regularization parameter introduced during the optimization. For these reasons, we set the value of $\alpha = 1000$.

3 FC patterns associated with nonneurophyisiological IMs

References

Parente, F. and Colosimo, A. (2020). Functional connections between and within brain subnetworks under resting-state. *Scientific Reports* 10, 3438

Rehman, N. and Aftab, H. (2019). Multivariate variational mode decomposition. *IEEE Transactions on Signal Processing* 67

Tzourio-Mazoyer, N., Landeau, B., Papathanassiou, D., Crivello, F., Etard,

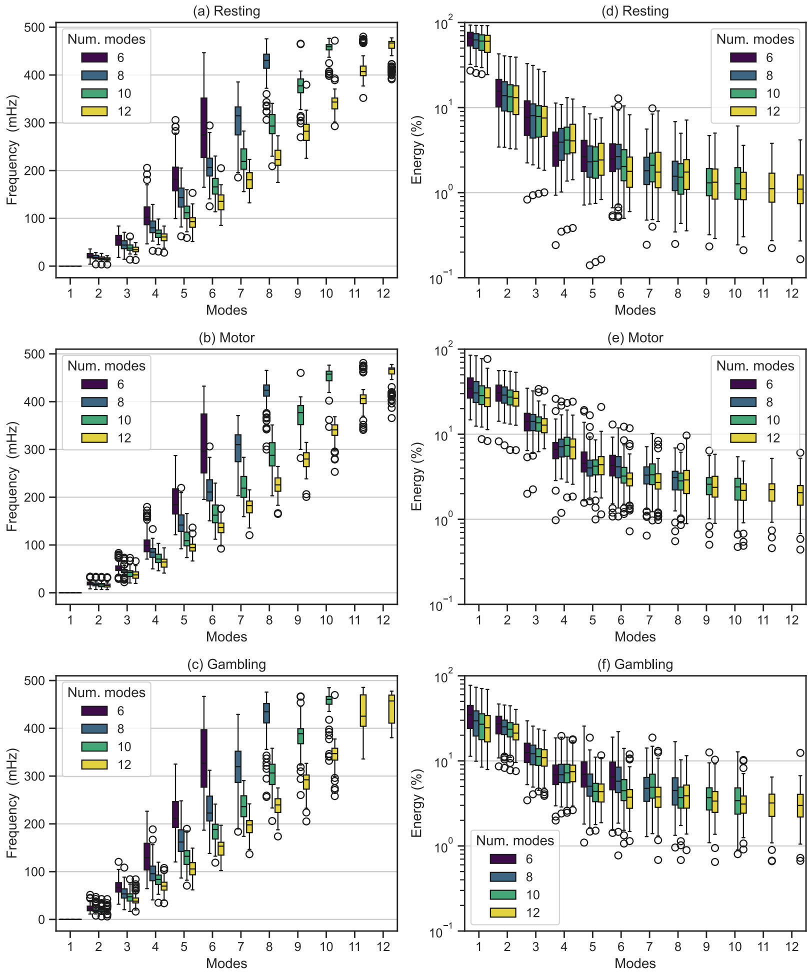

Figure 1: Frequency and energy distribution associated with each mode using MVMD for $\alpha = 1000$. The boxplots show the effect of total modes, K, for resting-state, motor, and gambling task fMRI experiments.

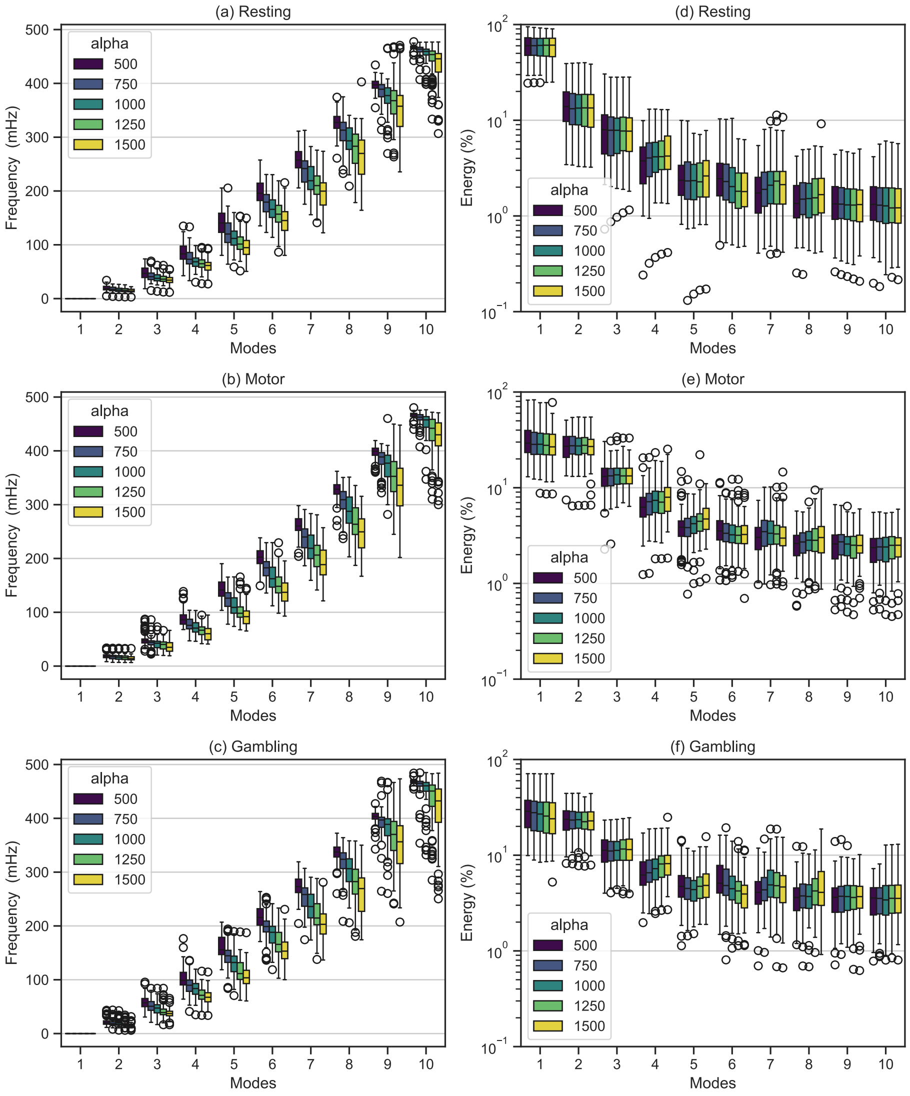

Figure 2: Frequency and energy distribution associated with each mode using MVMD for $K = 10$. Boxplots depict the effect of α among modes. The boxplots are for all the studied participants for resting-state, motor, and gambling task fMRI experiments.

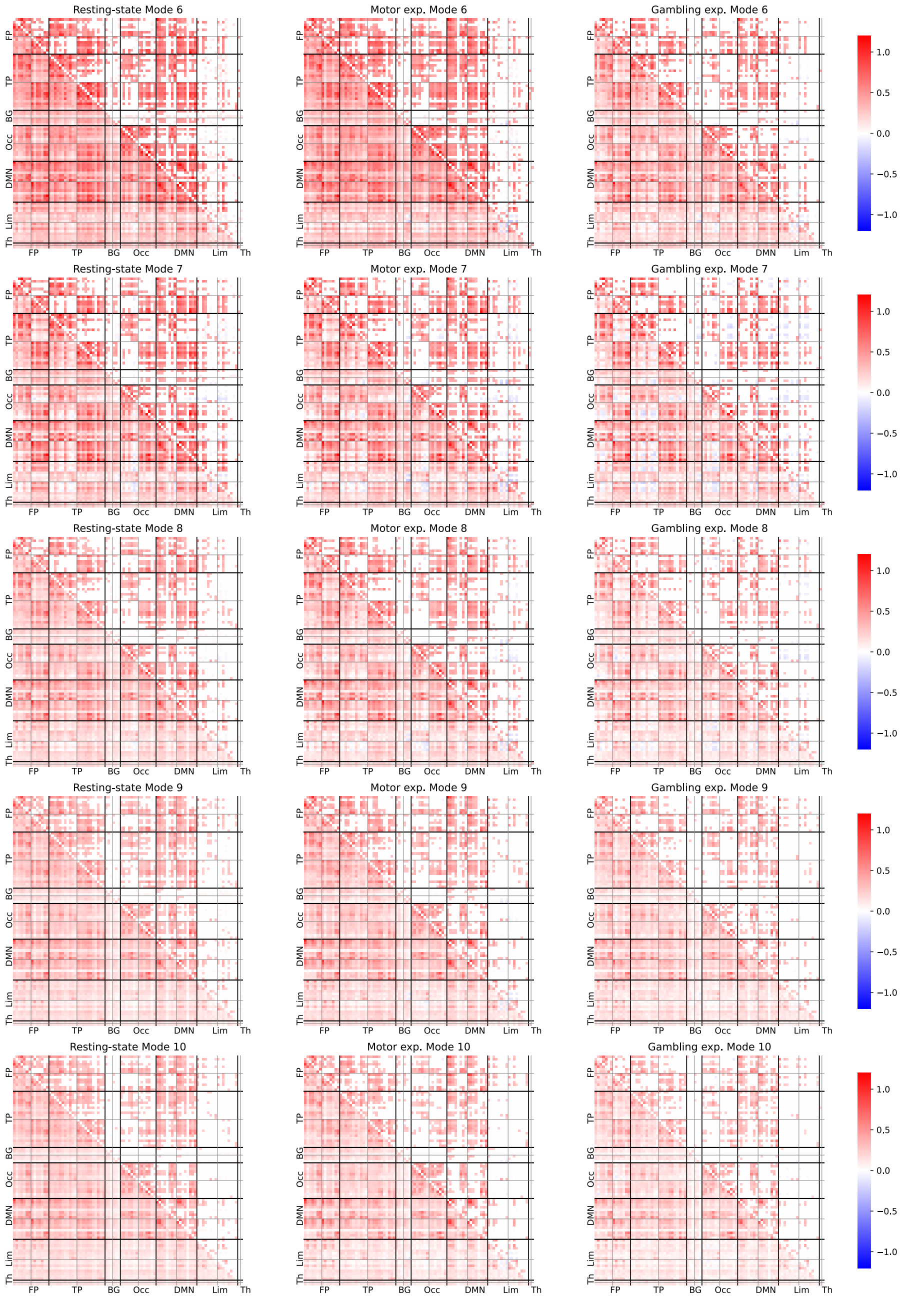

Figure 3: Average FC patterns for the high-frequency modes, i.e., IM 6, 7, 8, 9 and 10 for the three studied fMRI experiments. The FC patterns were estimated by averaging across 100 participants. Person's correlation coefficients were Fisher-Z transformed. The lower diagonal part shows all the averaged correlation coefficients. The upper diagonal only displays significant correlation coefficients compared to the null dataset from a permutation-based t-test corrected with a false positive rate adjusted to $p \leq 0.001$.

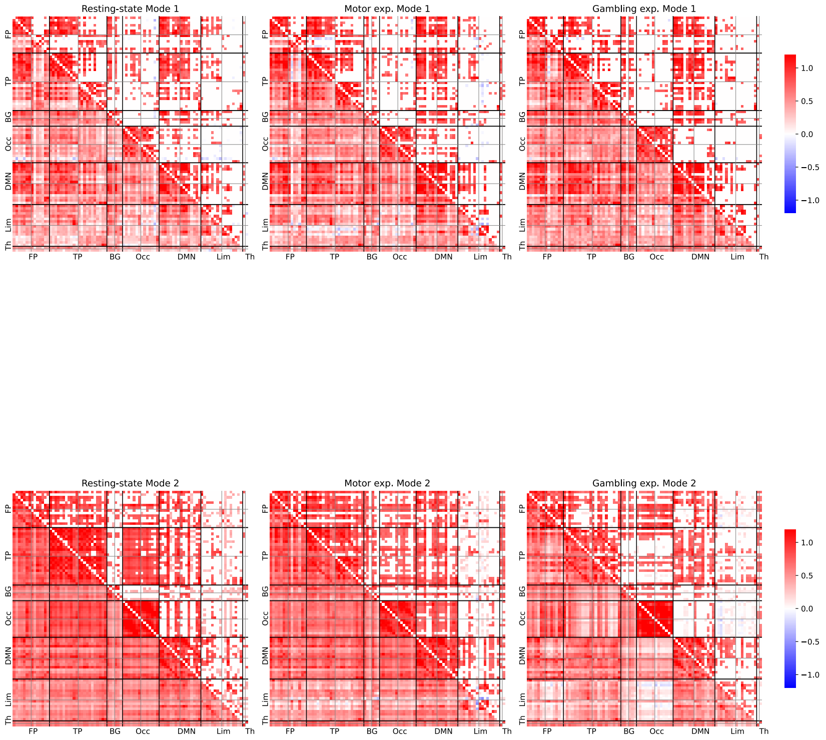

Figure 4: Average FC patterns for the first two IMs (IM1 and IM2) for the three studied fMRI experiments. The FC patterns were estimated by averaging across 100 participants. Person's correlation coefficients were Fisher-Z transformed. The lower diagonal part shows all the averaged correlation coefficients. The upper diagonal only displays significant correlation coefficients compared to the null dataset from a permutation-based t-test corrected with a false positive rate adjusted to $p \leq 0.001$.

O., Delcroix, N., et al. (2002). Automated anatomical labeling of activations in SPM using a macroscopic anatomical parcellation of the MNI MRI single-subject brain. *NeuroImage* 15, 273–289
